# The Evonik-Mainz Eye Care-Study (EMECS): Development of an Expert System for Glaucoma Risk Detection in a Working Population

**DOI:** 10.1371/journal.pone.0158824

**Published:** 2016-08-01

**Authors:** Jochen Wahl, Lorenz Barleon, Peter Morfeld, Andrea Lichtmeß, Sibylle Haas-Brähler, Norbert Pfeiffer

**Affiliations:** 1 Department of Ophthalmology, Helios Dr. Horst Schmidt Kliniken, Wiesbaden, Germany; 2 Department of Ophthalmology, University Medical Center, Johannes-Gutenberg-University, Mainz, Germany; 3 Department of Ophthalmology, Diakonissen Krankenhaus, Karlsruhe-Rüppurr, Germany; 4 Institute for Occupational Medicine of Cologne University, Köln, Germany; 5 Institute for Occupational Epidemiology and Risk Assessment of Evonik Industries, Essen, Germany; 6 Bundeswehrzentralkrankenhaus Koblenz, Abteilung X (Anästhesie, Intensivmedizin, Notfallmedizin, Schmerz), Koblenz, Germany; 7 Occupational Health, Evonik Industries, Hanau, Germany; Casey Eye Institute, UNITED STATES

## Abstract

**Purpose:**

To develop an expert system for glaucoma screening in a working population based on a human expert procedure using images of optic nerve head (ONH), visual field (frequency doubling technology, FDT) and intraocular pressure (IOP).

**Methods:**

4167 of 13037 (32%) employees between 40 and 65 years of Evonik Industries were screened. An experienced glaucoma expert (JW) assessed papilla parameters and evaluated all individual screening results. His classification into “no glaucoma”, “possible glaucoma” and “probable glaucoma” was defined as “gold standard”. A screening model was developed which was tested versus the gold-standard. This model took into account the assessment of the ONH. Values and relationships of CDR and IOP and the FDT were considered additionally and a glaucoma score was generated. The structure of the screening model was specified a priori whereas values of the parameters were chosen post-hoc to optimize sensitivity and specificity of the algorithm. Simple screening models based on IOP and / or FDT were investigated for comparison.

**Results:**

111 persons (2.66%) were classified as glaucoma suspects, thereof 13 (0.31%) as probable and 98 (2.35%) as possible glaucoma suspects by the expert. Re-evaluation by the screening model revealed a sensitivity of 83.8% and a specificity of 99.6% for all glaucoma suspects. The positive predictive value of the model was 80.2%, the negative predictive value 99.6%. Simple screening models showed insufficient diagnostic accuracy.

**Conclusion:**

Adjustment of ONH and symmetry parameters with respect to excavation and IOP in an expert system produced sufficiently satisfying diagnostic accuracy. This screening model seems to be applicable in such a working population with relatively low age and low glaucoma prevalence. Different experts should validate the model in different populations.

## Introduction

Glaucoma is one of the leading causes of visual impairment and blindness worldwide [1]. It is a heterogeneous group of diseases affecting the optic nerve head (ONH). Primary open angle glaucoma (POAG) is the most frequent entity in most ethnicities.

The pathophysiological processes leading to glaucoma are not completely understood. Increased intraocular pressure is a main risk factor for glaucoma. Individuals with high intraocular pressure (IOP) may develop glaucoma with ONH damage and visual field defects (glaucoma cases). However, thresholds may be heterogeneous across a population of individuals above which IOP may cause damage. Thus, there are subjects with high IOP and no glaucoma (ocular hypertension cases) on the one hand, and patients with normal IOP and severe ONH damage (normal tension glaucoma cases) on the other hand [2,3].

It has been shown that about 20% to 60% of the nerve fibres are damaged before visual field defects occur [4]. In the initial stage of the disease, there are no symptoms. Knowledge about glaucoma and its risks in the general population is poor [5]. In Germany, health insurance companies do not reimburse medical prevention examinations (preventive medical care) such as measurement of the IOP or photos of the ONH. In many cases, POAG is diagnosed in a late stage when ONH damage and visual field defects are already present. It can be assumed that a large proportion of–glaucoma patients are undiagnosed and hence untreated [6].

The prevalence of POAG increases with age and doubles per decade [7]. A screening program in the working population might reach individuals in whom timely therapy may prevent later loss of visual function.

On the basis of legal requirements, companies in Germany offer occupational health supervision and medical exams to their employees to detect functional disorders which may influence the employee’s ability to work in their jobs. These exams include the so called G-examinations, G-37 for visual display unit (VDU) workplaces (to performed at least once within 3 years for ages > 40 years) and G-25 for driving, controlling and monitoring work (repeated every 2nd to 3rd year for ages > 40 years and every first to 2nd year for ages > 60 years) [8].

Based on these regulations, it seemed to be useful to add examinations for glaucoma screening within a trial. This approach has been described for a more general ophthalmological setting and cooperation with the occupational health sector in a large German chemical company [8]

We used this setting to perform a glaucoma screening within the working population. The primary aim of this investigation was to develop an expert system for efficient screening. The basic data was obtained by an expert’s evaluation of digital images of the optic nerve head (ONH), the intraocular pressure (IOP) and perimetry (frequency doubling technology, FDT). He used these data to identify glaucoma suspects. An algorithm was designed to simulate the expert’s decision process. The second aim was to test whether a screening program for glaucoma is feasible within the setting of an active working population based on differing screening parameters.

## Methods

Recruitment of study subjects, ophthalmological procedures and basic data are described elsewhere [8]. In brief, 4183 out of 13037 employees older than 40 years were examined at 13 sites of Evonik Industries between June 2007 and March 2008. For this investigation, focussing on glaucoma screening, 16 subjects had to be excluded due to missing FDT values.

The authors intended to use simple routine eye examinations which are easy to handle and can be performed by assistance staff working in the occupational health departments. For that reason, only non-contact examinations were used. The assistance staff performed non-contact IOP measurements, FDT and non-mydriatic fundus photography. The staff was trained in advance by experts in the department of ophthalmology at Johannes Gutenberg University Mainz, Germany.

The collected data was handed over electronically to an experienced glaucoma expert (JW) who assessed papilla parameters and evaluated all individual screening results. This procedure guaranteed that the collection of the data could be performed by the staff at the Evonik sites and the medical evaluation later on by an ophthalmological expert at the University.

The routine eye examinations within occupational medicine (G25, G37) were described elsewhere [8]. Here we confined ourselves to the add-on examinations for glaucoma screening.

### Tonometry

The intraocular pressure (IOP) was measured using a non-contact tonometer (AT 555, Reichert Ophthalmic Instruments, Depew, NY, USA). Intraocular pressure (IOP) was measured 3 times, starting with the right eye, and the mean of these 3 measurements was used for further statistical analysis.

### Perimetry

For perimetric examination, we used Frequency Doubling Technology (FDT) (Humphrey® FDT, Carl Zeiss Meditec, Jena, Germany) for both eyes, starting with the right eye. Due to screening conditions, the FDT screening program C-20-5 with 17 screen patterns was chosen. We used the estimated probability value P, rendered by this program, for classification (P ≥ 5%: within normal limits; P < 5%: mild relative loss; P < 2%: moderate relative loss, P < 1%: severe loss).

We classified FDT C20-5 for each eye by our own classification system, as pathological if

≥ 3 screen patterns had a P-value < 5%, or if
≥ 2 screen patterns had a P- value < 2%, or if
≥ 1 screen pattern had a P-value < 1%, or if
≥ 1 screen pattern had a P-value < 2% plus ≥ 1 screen pattern had a P-value < 5%, or if
≥ 2 connected screen patterns had a P-value < 5%.

Furthermore, we defined for each eye, that a FDT C20-5 result was not reliable if a fixation error (FE) or a false positive error (FPE) was present more frequent than in 1 out of 3 catch trials (> 1/3). After evaluation of FDT C20-5 for each eye, each participant was categorized as either without or with pathological FDT-findings.

For the subject we defined the FDT as normal based on the data of both eyes as follows:

1. Subject without pathological FDT-findings, if:
  1. both eyes had no pathological FDT or
  2. one eye had no pathological FDT and the other eye revealed no reliable results or no results available for the other eye
2. With pathological FDT- findings, if:
  1. both eyes had pathological FDT or
  2. one eye had a pathological FDT and the other eye had no pathological FDT or
  3. one eye had a pathological FDT and the other side rendered no reliable results or no results available for the other eye
  The glaucoma expert evaluated a pathological FDT as typical or not typical for glaucoma, e.g. whether the pathological FDT fields were appropriately connected.
3. We excluded participants from this analysis, if both eyes revealed a non reliable FDT.

### Fundus Photography

A 45° fundus photography was performed with a non-mydriatic retinal camera (Non-Mydriatic Retinal Camera CR-DGi, Canon Inc., Tokyo, Japan).

An evaluation of the fundus photography with respect to glaucoma was performed by a glaucoma expert (JW) at the Department of Ophthalmology, University Medical Center, Johannes-Gutenberg-University, Mainz, Germany. The optic disc was evaluated by size, cup-disc-ratio, ISNT-rule, morphology of excavation, disc haemorrhages and asymmetry between optic disc of right and left eyes [10].

Other findings (e.g. drusinosis, edema or bleedings) were noted. In case of pathological findings a recommendation was given to consult an ophthalmologist.

### Glaucoma classification of the participants based on the expert assessment

Immediately after the examinations the expert received the results and classified the subjects into three categories: no glaucoma, possible glaucoma and probable glaucoma. ‘Glaucoma suspect’ was defined as either possible or probable glaucoma.

The expert generated his judgement on the base of the results of the single examinations, although the way in which they were combined was not a priori defined. The judgement of the expert was taken as ‘gold standard’, i.e. as a reference in subsequent comparisons.

### The screening algorithm for glaucoma diagnosis

The authors constructed an algorithm, which was supposed to simulate the glaucoma expert’s decision process. An expert meeting was held at the Johannes Gutenberg University to derive appropriate scores. The intention was to focus on the morphology of the ONH and not to put too much weight on the IOP. The input data to the algorithm consisted of the following information generated during the expert’s assessment for each eye using the fundus photographies. According to the opinion of the expert group photographies of the optic nerve head are the simplest way to represent the clinical examination. Laser scanning or polarimetric techniques were available but they did not seem to be reliable as when used alone to examine the optic nerve head.

- Assessment of the ONH with respect to the ISNT-rule (inferior-superior-nasal-temporal rule) [10]
- size of the ONH–categorized in small, medium, large (crude estimate)
- horizontal cup to disc ratio (CDR)
- vertical CDR
- reduction in nerve fibre layer
- notch in nerve fibre layer

We defined CDR_max_ as the maximum of the vertical and horizontal CDR.

**Fig 1:**
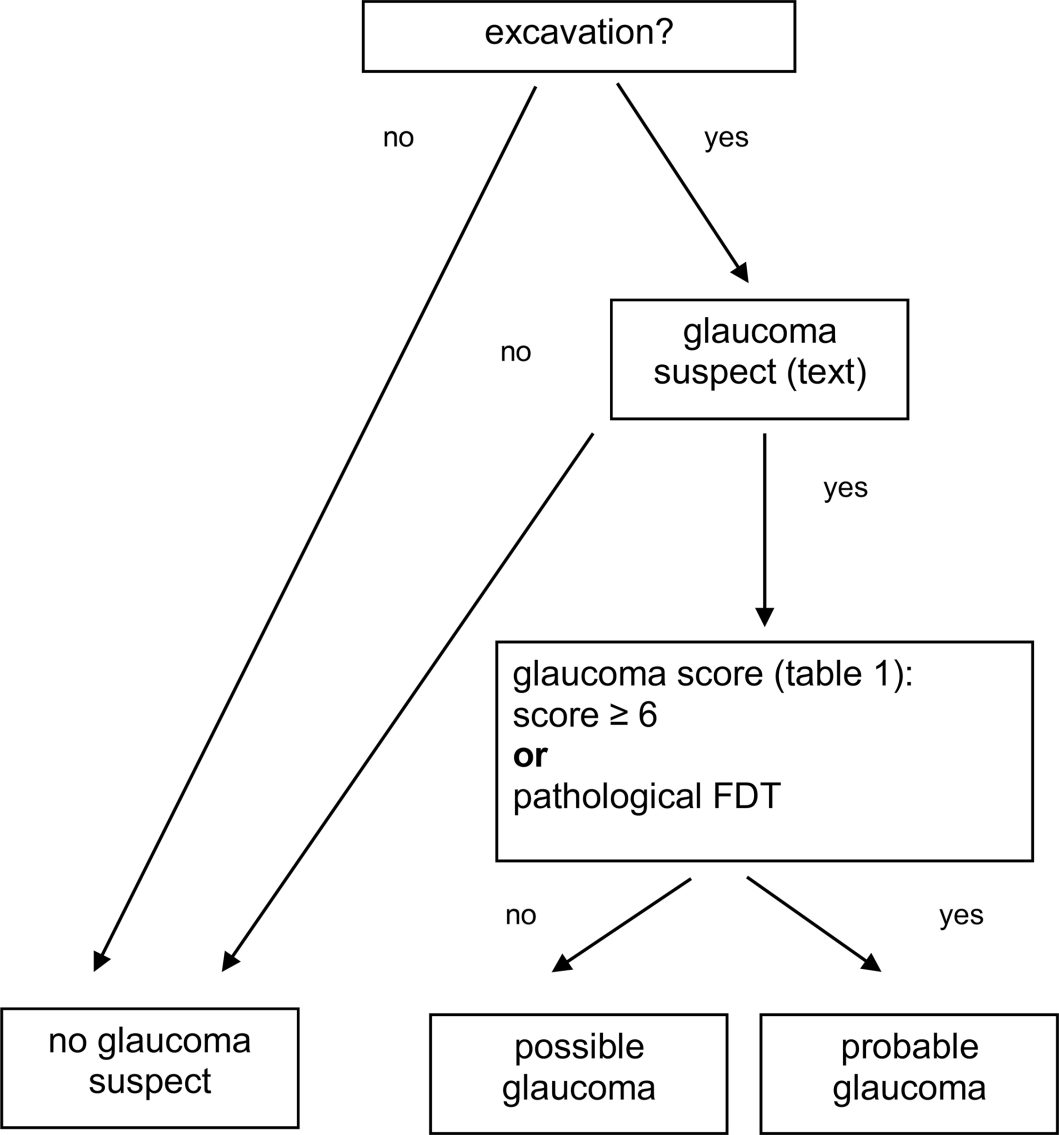
Algorithm for the classification of single eyes into ‘no glaucoma suspect’, ‘possible’ and ‘probable’ glaucoma suspect. Glaucoma suspect is defined in the text, for the glaucoma score definition see Table 1.

Furthermore, we used the outcome of the FDT examination and IOP measurements (see Table 1 and Fig 1 for details).

**Table 1:** Calculation of a glaucoma score for the classification into possible or probable glaucoma. The score was defined as the sum of the points. Calculation was performed for each eye separately.

Condition	points
IOP > 28 mm Hg	1 point
IOP difference* ≥ 2 mm Hg	1 point
CDR_max_ difference* ≥ 0.1	1 point
Excavation of ONH	
- nerve fibre layer slightly reduced	1 point
- notch in nerve fibre layer	2 point
- complete loss of nerve fibre layer	6 point
ISNT-rule not respected	1 point
Optic disc haemorrhage	1 point
CDRmax > 0.6 in small ONH or	1 point
CDRmax > 0.7 in medium-sized ONH or	
CDRmax > 0.8 in large ONH	
	sufficient scores
*possible glaucoma*	Ʃ > 1 points
*probable glaucoma*	*Ʃ > 6 points*

*in comparison to the partner eye

Using these data the screening algorithm was defined as follows to diagnose single eyes as glaucoma suspects:

- If there was no excavation (horizontal and vertical CDR equal 0) the subject was classified as ‘no glaucoma suspect’.
- If there was an excavation (horizontal or vertical CDR not equal 0) an evaluation of the excavation data followed. An eye is classified ‘glaucoma suspect’, if at least one of the following conditions is fulfilled:
  - ISNT-rule not respected
  - CDR_max_ increased taking ONH size into account (large ONH: CDR_max_ > 0.8; medium-sized ONH: CDR_max_ > 0.7; small ONH: CDR_max_ 0.6)
  - notch in nerve fiber layer
  - haemorrhage at rim of optic nerve head
  - in comparison to the partner eye: CDR_max_ more than 0.2 larger and IOP more than 3 mm higher

Otherwise the eye was classified as ‘no glaucoma suspect’.

If classified as ‘glaucoma suspect’, a risk score was created depending on the possible state of glaucoma damage in relation to the size of the ONH, the IOP as a risk factor, an asymmetry between the ONHs and higher IOP in the eye with the ONH with the larger CDR_max_, rim bleedings at the ONH, and the distribution of the nerve fiber rim (Table 1).

The score was constructed with the intention that a range between one and 6 points indicates ‘possible glaucoma’ and equal to or more than 6 points indicate ‘probable glaucoma’.

After the expert group identified initial parameters a part of the scoring system (symmetry parameters) was optimized by fitting the algorithm repeatedly to the data. To do so, the parameters of IOP difference and asymmetry in the CDR_max_ were calculated in several passes with different values in order to optimize the screening procedure in comparison to the results of the glaucoma expert (when we choose as criteria an IOP difference of at least 2 mm HG and an asymmetry of at least 0.1 in CDR we calculated almost the same number of individuals as the expert classified as ‘possible glaucoma’ and ‘probable glaucoma’).

If the eye was classified as ‘glaucoma suspect‘, and the FDT was pathological, the eye was also classified as ‘probable glaucoma’ independently of the result from the score. All other glaucoma eyes were classified as ‘possible glaucoma’. An overview of the decision process for single eyes is given in Fig 1.

To diagnose an individual based on the results of both eyes, we constructed a cross tabulation with the diagnosis for the right eye on one side and for the left eye on the other side (Table 2).

**Table 2:** Cross tabulation for diagnosing individuals on categorization of left and right eyes. Numbers are defined in the marginals and final diagnoses of the individuals are given in cells.

right eye →		no glaucoma suspect and visual field defect of different genesis	OHT, and visual field defect of different genesis	no glaucoma suspect, normal result	OHT	possible glaucoma with IOP > 21 mm Hg	Possible normal tension glaucoma	probable glaucoma with IOP > 21mmHg	probable normal tension glaucoma	OHT and FDT not defined	no glaucoma suspect and FDT not defined
left eye ↓		1	2	3	4	5	6	7	8	9	10
no glaucoma suspect and visual field defect of different genesis	1	1	2	1	4 + visual field defect in the other eye of different genesis	5+ visual field defect in the other eye of different genesis	6+ visual field defect in the other eye of different genesis	7+ visual field defect in the other eye of different genesis	8+ visual field defect in the other eye of different genesis	2	1
OHT, and visual field defect of different genesis	2	2	2	2	2	5 + visual field defect in the other eye of different genesis	5 + visual field defect in the other eye of different genesis	7+ visual field defect in the other eye of different genesis	7+ visual field defect in the other eye of different genesis	2	2
no glaucoma suspect, normal result	3	1	2	3	4	5	6	7	8	4	3
OHT	4	4 + visual field defect in the other eye of different genesis	2	4	4	5	5	7	7	4	4
possible glaucoma with IOP > 21 mm Hg	5	5 + visual field defect in the other eye of different genesis	5 + visual field defect in the other eye of different genesis	5	5	5	5	7	7	5	5
possible normal tension glaucoma	6	6 + visual field defect in the other eye of different genesis	5 + visual field defect in the other eye of different genesis	6	5	5	6	7	8	5	6
probable glaucoma with IOP > 21 mm Hg	7	7 + visual field defect in the other eye of different genesis	7 + visual field defect in the other eye of different genesis	7	7	7	7	7	7	7	7
probable normal tension glaucoma	8	8+ visual field defect in the other eye of different genesis	7+ visual field defect in the other eye of different genesis	8	7	7	8	7	8	7	8
OHT and FDT not defined	9	2	2	4	4	5	5	7	7	exclusion	exclusion
no glaucoma suspect and FDT not defined	10	1	2	3	4	5	6	7	8	exclusion	exclusion

OHT = ocular hypertension. Categories 9 and 10 report on the overall decision if FDT was not measurable or FE ≥ 2/3 or FPE ≥ 2/3.

We used a cut point of 21 mm Hg to define on ocular hypertension (OHT). We like to emphasize that only those participants were diagnosed as ocular hypertensive in the left (or right) eye, if the mean IOP was higher than 21 mmHg in the left (or right) eye and if the left (or right) eye was classified as ‘no glaucoma suspect’ [9]. For the definition of ‘no glaucoma suspect’ see below. The definition of OHT based on data of both eyes is described in Table 2.

### Alternative Screening Models

Since the algorithm includes findings from the fundus photographies, the authors tested if more simple models could be applied with an equal or sufficient diagnostic accuracy.

#### Screening model 1

In screening model 1 a subject was defined as glaucoma suspect based on data of both eyes if at least one eye had IOP > 21 mm Hg **or** the FDT was pathological. This model was expected to be rather sensitive but not specific.

#### Screening model 2

In screening model 2 a subject was defined as glaucoma suspect based on the data of both eyes if at least one eye had IOP > 21 mm Hg **and** the FDT was pathological. This model was expected to be less sensitive but of higher specificity than model 1, possibly identifying probable glaucoma with higher accuracy.

#### Screening model 3

Screening model 3 as the simplest model mostly used by non-ophthalmologists is to consider individuals as glaucoma suspects if at least one eye had IOP > 21 mm Hg.

In all three alternative screening models the eye with the more severe classification determined the classification of the individual. If one eye could not be evaluated but the other was classified as ‘no glaucoma suspect’, we categorized the subject as ‘ glaucoma suspect’.

The testing, whether FDT alone could deliver a reliable model was not performed because too many other ophthalmological reasons apart from glaucoma could cause pathological results. All individuals with a pathological FDT are part of screening model 1.

### Data Protection Issues

All individual data of each participant were encrypted with a barcode at each local Department of Occupational Health of Evonik Industries (pseudonomization). Immediately after the medical examination all recorded data, collected under each related barcode, were sent to the Department of Ophthalmology at Mainz University for evaluation. Afterwards the results and recommendations were sent back to the corresponding local Department of Occupational Health of Evonik Industries, where a re-identification of the personal data was performed. Pseudonominized individual data were transferred to the Institute for Occupational Epidemiology and Risk Assessment of Evonik Industries for analyses.

### Ethics Statements

All participants gave written informed consent before entry into the study. No open personal data were available at the University of Mainz and the additional medical examination was covered by the regulations of occupational medicine. All examinations were non-invasive. The study protocol and data protection procedures were submitted to and accepted by the data protection office of DEGUSSA (K. Gowig, Head of Department of Data Protection, RAG-Beteiligungs-AG, Essen Germany). At that time this was the responsible institutional review board. This review board approved the study protocol. In the meantime, DEGUSSA became part of Evonik Industries. No new ethics committee approval was obtained or deemed necessary after this change.

All study procedures adhered to the recommendations of the Declaration of Helsinki.

### Statistical analysis

The screening algorithm and each of the screening models 1–3 were statistically compared with the judgement of the ophthalmologist (‘gold standard’).

We determined the following statistics to evaluate diagnostic accuracy: specificity, sensitivity, positive predictive value, negative predictive value, positive likelihood ratio, negative likelihood ratio [11–14].We used the delta method to calculate 95%-confidence intervals (95%-CI) for percentages using the logit transformation [15,16], [17]. We calculated Agresti-Coull intervals additionally [17] to check whether the logit-intervals were appropriate. We calculated pre-test and post-test odds and prevalences based on the positive and negative likelihood ratios [13]. In addition, we inverted the negative likelihood ratio for ease of comparison with the positive likelihood ratio.

Diagnostic odds ratios were calculated to test the null hypothesis of no association and to measure the degree of dependence between screening procedures and expert judgement (see S1 Appendix) [18]. Prevalence odds ratios can be used as statistics approximating relative risks if the baseline risk is below 10% [19]. Moreover, we calculated kappa statistics to estimate the agreement between screening method and expert evaluation [20,21].

See the S1 Appendix for definition of the terms and the relationships used. S1 Appendix gives a suggestion how kappa values may be interpreted.

We estimated “numbers needed to screen” according to the procedures used to calculate “numbers needed to treat” [22]. We applied this statistic in cost calculation and determination of effectiveness of the screening procedures.

Statistical analyses were performed with Stata 10 (StataCorp LP, College Station, Texas, USA). A significance level of 5% was chosen.

## Results

### Demographic description and basic data

A detailed demographic description of the study group is given in the publication by Barleon et al. [8]

For this investigation, focussing on glaucoma screening, 16 subjects out of 4183 had to be excluded due to missing FDT values. In principle, among these 16 subjects, glaucoma suspects could have been identified based on glaucoma score ≥ 6 (Fig 1). Because this did not occur, we excluded these subjects from the analyses. Therefore, 4167 employees were included in the analyses. An overview on the age and sex distribution is given in Table 3.

**Table 3:** Age and sex distribution.

age class	male	female	total
40–44	869	328	1197
45–49	999	280	1279
50–54	830	201	1031
55–59	459	135	594
60–65	54	12	66
Total	3211	956	4167

### Intraocular pressure (IOP) (right n = 4166, left n = 4164)

The mean IOP was 16.1 ± 3.4 mmHg, both in the right and in the left eye, with a range from 2 to 41 mmHg in the right and from 8 to 42 mmHg in the left eye. 358 subjects (8.6%) had an IOP > 21 mmHg in at least one eye and 177 (4.2%) had an IOP > 21 mmHg in both eyes.

29 out of 4167 subjects were already treated for glaucoma with a mean IOP in the right eye of 17.9 ± 3.9 mmHg (range from 10 to 29 mmHg) and in the left eye of 19.5 ± 4.0 mmHg (range from 12 to 28 mmHg). Among these 29 subjects were 9 subjects with an IOP greater than 21 mmHg in at least one eye.

### Visual field and IOP

Due to missing or unreliable FDT perimetry for both eyes, 16 subjects out of 4183 had to be excluded from all future analyses. A reliable FDT perimetry could be performed for 4121 subjects (98.9%) in the right and 3978 (95.5%) subjects in the left eye. 3995 subjects (95.9%) showed no pathological finding in the right and 3810 (91.4%) in the left eye. 126 subjects (3.0%) showed pathological findings in the right and 168 subjects (4.0%) in the left eye. 46 subjects (1.1%) had missing FDT or no reliable data in the right and 189 (4.5%) in the left eye.

3932 individuals (94.4%) showed reliable results in both eyes. 3705 (88.9%) had no and 54 (1.3%) had pathological findings in both eyes. 173 (4.2%) had pathological findings in one eye and no pathological findings in the other. Thereof had 112 (2.7%) no pathological findings in the right but in the left eye and 61 (1.5%) no pathological findings in the left but in the right eye (Table 4).

**Table 4:** Cross-classified table for all FDT results (n = 4167).

		FDT perimetry left eye			
		without pathological findings	with pathological findings	missing or not reliable FDT data	Total
**FDT perimetry right eye**	**withou**t pathological findings	3705	112	178	**3995**
	**with** pathological findings	61	54	11	**126**
	missing or not reliable FDT data	44	2	0	**46**
**Total**		**3810**	**168**	**189**	**4167**

Table 5 reports on the cross tabulation of IOP measurements and FDT results.

**Table 5:** Distribution of elevated IOP and pathological FDT, based on both eyes.

	Pathologic FDT		
IOP	No	Yes	Total
≤ 21 mm Hg	3589	219	3808
> 21 mm Hg	337	21	358
missing	1	0	1
total	3927	240	4167

3589 subjects had no pathological findings in the FDT and an IOP of ≤ 21 mm Hg. 337 (8.1%) subjects had no pathological findings in the FDT and an IOP > 21 mm HG. 219 (5.3%) persons had a conspicuous FDT and an IOP ≤21 mm. Only 21 (0.5%) had an IOP > 21 mm Hg and pathological findings in the FDT. 1 person (0.02%) had a missing value in the IOP measurement but no pathological findings in the FDT.

### Prevalence of glaucoma suspects and diagnostic accuracy of the algorithm as a screening method

According to the assessment by the ophthalmologist, 98 (2.35%) subjects were classified as ‘possible glaucoma’ and 13 (0.31%) as ‘probable glaucoma’. In total, 111 employees (2.66%) were classified as glaucoma suspects. Subjects not yet diagnosed to have glaucoma or not knowing about such a diagnosis received the recommendation to consult an ophthalmologist.

The classification by the screening algorithm revealed 104 (2.5%) employees as ‘possible glaucoma’ and 12 (0.29%) as ‘probable glaucoma’. Thus, 116 employees (2.79%) were classified as glaucoma suspects. Overviews of the findings on diagnostic accuracy are given in Tables 6 and 7.

**Table 6:** Comparison of different screening models with respect to the different statistical parameters, glaucoma suspects. The assessment of the ophthalmologist was defined as “gold standard”. He appraised 4056 (97.34%) as “no glaucoma suspects” and 111 (2.66%) as “glaucoma suspects”.

	No Glaucoma Suspect	Glaucoma Suspect	Sensitivity %	Specificity %	Positive Predictive Value %	Negative Predictive Value %	Positive Likelihood Ratio	Negative Likelihood Ratio	Inverse Negative Likelihood Ratio	Diagnostic Odds Ratio	Kappa (Std Err)
Algorithm	4051 (97.22%)	116 (2.79%)	83.78 (75.72–89.54)^†^	99.43 (99.15–99.62)^†^	80.17 (71.92–86.45)^†^	99.56 (99.30–99.72)^†^	147.8	0.16	6.13	905.60 (472.9–1735.7)^†^	0.8143 (0.0155)
Screening 1 IOP > 21 mm Hg or FDT pathological	3589 (86.13%)	578 (13.87%)	65.77 (56.48–73.98)^†^	87.55 (86.50–88.53)^†^	12.63 (10.16–15.59)^†^	98.94 (98.55–99.23)^†^	5.28	0.39	2.56	13.51 (9.0–20.2)^†^	0.1750 (0.0109)
Screening 2 IOP > 21 mm Hg and FDT pathological	4146 (99.5%)	21 (0.5%)	6.31 (3.04–12.64)^†^	99.65 (99.42–99.80)^†^	33.33 (16.79–55.33)^†^	97.49 (96.97–97.93)^†^	18.3	0.94	1.06	19.4 (7.7–49.2)^†^	0.0984 (0.0112)
Screening 3* IOP > 21 mm Hg	3808 (91.38%)	358 (8.59%)	55.45 (46.08–64.45)^†^	92.68 (91.83–93.44)^†^	17.04 (13.49–21.29)^†^	98.71 (98.30–99.03)^†^	7.57	0.48	2.08	15.8 (10.6–23.4)^†^	0.2296 (0.0129)

* 1 missing value

^†^ 95% confidence intervals

**Table 7:** Comparison of different screening models with respect to the different statistical parameters, probable glaucoma. The assessment of the ophthalmologist was defined as “gold standard”. He appraised 4154 (99.69%) as “not probable glaucoma” and 13 (0.31%) as “probable glaucoma”.

	Not Probable Glaucoma	Probable Glaucoma	Sensitivity %	Specificity %	Positive Predictive Value %	Negative Predictive Value %	Positive Likelihood Ratio	Negative Likelihood Ratio	Inverse Negative Likelihood ratio	Diagnostic Odds Ratio	Kappa (Std Err)
Algorithm	4155 (99.71%)	12 (0.29%)	84.62 (54.94–96.12)^†^	99.98 (99.82–99.99)^†^	91.67 (58.68–98.84)^†^	99.95 (99.81–99.99)^†^	3514.92	0.1539	6.50	22841.5 (1927.6–270667)^†^	0.8796 (0.0155)
Screening 1 IOP > 21 mm Hg or FDT pathological	3589 (86,13%)	578 (13.87%)	1	86.40 (85.32–87.41)^†^	2.25 (1.31–3.83)^†^	1	7.35	0	.	.	0.0381 (0.0042)
Screening 2 IOP > 21mm Hg and FDT pathological	4146 (99.50%)	21 (0.50%)	15.38 (3.87–45.06)^†^	99.54 (99.28–99.71)^†^	9.52 (2.39–31.13)^†^	99.73 (99.52–99.85)^†^	33.64	0.85	1.18	39.6 (8.2–190.7)^†^	0.1142 (0.0151)
Screening 3* IOP > 21mm Hg	3808 (91.38%)	358 (8.59%)	61.54 (34.36–83.02)^†^	91.57 (90.69–92.38)^†^	2.23 (1.12–4.40)^†^	99.87 (99.68–99.95)^†^	7.30	0.42	2.38	17.4 (5.7–53.4)^†^	0.0373 (0.0055)

* 1 missing value

^†^ 95% confidence intervals

The sensitivity of the screening algorithm to detect a glaucoma suspect (sum of possible and probable glaucoma) was 0.84 (95% Cl: 0.76–0.90). The specificity of the algorithm was 0.99 (95% CI: 0.99–1.0).

The sensitivity of the algorithm to detect a probable glaucoma (highly glaucoma suspect) was 0.85 (95% CI: 0.56–0.96). The specificity of the algorithm was 1.0.

The positive predictive value (PPV) of the algorithm to detect a glaucoma suspect was 0.80 (95% CI: 0.72–0.86) and the negative predictive value (NPV) was 1.0 (95%CI: 0.99–1.0).

The positive predictive value (PPV) of the algorithm to detect probable glaucoma was 0.92 (95% CI: 0.59–0.99) and the negative predictive value (NPV) was 1.0.The odds-ratio to detect a glaucoma suspect was 906 (95% CI: 473–1736). In probable glaucoma suspects the odds ratio was 22842 (95% CI: 1928–2707000).

With respect to glaucoma suspects the positive likelihood-ratio was 147.8, the negative likelihood ratio was 0.16, thus the inverse negative likelihood ratio was 6.13. With respect to probable glaucoma the positive likelihood ratio was 3514.9, the negative likelihood ratio was 0.1539 (inverse negative likelihood ratio = 6.5).

### Other Screening models

The diagnostic accuracy measures derived for the different screening models 1–3 are summarized in Table 6 for glaucoma suspects (the sum of possible and probable glaucoma), and for probable glaucoma in Table 7.

### Expert decisions and screening procedures in treated glaucoma patients

Table 8 shows the joint distribution of glaucoma suspects according to the expert and the algorithm by existing medication for glaucoma. 35 of the subjects were already treated as glaucoma patients. 9 of those were identified by the algorithm as glaucoma suspects, and nine were classified as glaucoma suspects by the expert. Eight of the already treated individuals were categorized as suspects by the expert and the algorithm. One was classified as glaucoma suspect by the expert, but not by the algorithm. In addition, one was classified as glaucoma suspect by the algorithm, but not by the expert. Thus, 26 of the already treated individuals were not classified as glaucoma suspects by the expert and 102 of those who were classified as glaucoma suspects were not treated. The corresponding numbers for the algorithm were 26 and 107. Furthermore, 111 were classified as glaucoma suspects by the expert. Out of those only 9 subjects were treated with glaucoma medication. The corresponding numbers for the algorithm were 116 and 9. 17 subjects classified as glaucoma suspects by the expert that were not identified by the algorithm and were not on medication already.

**Table 8:** Subjects treated with glaucoma medication and relation to the algorithm’s and to expert’s decision on the prevalence of glaucoma suspects.

Algorithm		No		Yes	
Expert		No	Yes	No	Yes
Treated with glaucoma medication					
	No	4008	17	22	85
	Yes	25	1	1	8
	Total	4033	18	23	93

Table 9 shows the joint distribution of probable glaucoma suspects according to the expert and the algorithm by existing medication for glaucoma. Six out of the 35 treated subjects were classified by the algorithm as probable glaucoma suspects, and seven were classified as probable glaucoma suspects by the expert. Six of the already treated individuals were identified by the expert and the algorithm as probable glaucoma. One was classified as probable glaucoma suspect by the expert, but not by the algorithm. Therefore, 28 of the already treated individuals were not classified as probable glaucoma suspects by the expert and six of those who were classified as probable glaucoma suspects were not treated. The corresponding numbers for the algorithm were 29 and six. Furthermore, 13 were classified as probable glaucoma suspects by the expert. Out of those only 7 subjects were treated with glaucoma medication. The corresponding numbers for the algorithm were 12 and 6. Only one subject classified as probable glaucoma suspect by the expert was not identified by the algorithm as such and was not on medication already.

**Table 9:** Subjects treated with glaucoma medication and relation to the algorithm’s and to expert’s decision on the prevalence of probable glaucoma suspects.

Algorithm		No		Yes	
Expert		No	Yes	No	Yes
Treated with glaucoma medication					
	No	4125	1	1	5
	Yes	28	1	0	6
	Total	4153	2	1	11

### Number needed to screen

Among the 13 suspects classified as probable glaucoma (high risk glaucoma suspects) 7 had not previously been diagnosed as having glaucoma (Tables 9 and 10).

**Table 10:** Number needed to screen.

	probable glaucoma detected	no probable glaucoma
Without screening	6	4161
Screening	13	4154

We calculated how many individuals are needed to be screened by the expert in order to detect a so far undiscovered subject with probable glaucoma.

Calculation of the absolute risk reduction (ARR)

$$ARR=\frac{13}{4167}-\frac{6}{4167}=\frac{7}{4167}=0.0168$$

Calculation of the number needed to screen (according to the concept of ‘number needed to treat, NNT)

$$NNT=\frac{1}{ARR}=\frac{4167}{7}=595.3$$

This means that 595 individuals have to be screened to detect one probable glaucoma case who has not previously been diagnosed as having glaucoma.

According to Table 9, 12 subjects were classified as probable glaucoma suspects by the algorithm. Out of those only 6 subjects were treated with glaucoma medication. Thus, the absolute risk reduction based on the algorithm is ARR = 0.00144 and we get NNT = 694.5.

Furthermore, 111 were classified as glaucoma suspects by the expert (Table 8). Out of those only 9 subjects were treated with glaucoma medication. The absolute risk reduction ARR was 0.0245 and NNT = 40.9. The corresponding numbers for the algorithm were 116 and 9 (ARR = 0.0257, NNT = 38.9).

### Cost-Calculation

In a cost-calculation-model it was estimated that a total amount of 511,000 € for the whole study was spent (Table 11). Thus, a rough estimate reveals that about

$$\frac{511000\;€}{4183}\approx 122\;€$$

were spent for the examination of a single employee.

Thus, about 73000 €

$$\frac{511000\;€}{4183}\times 595.3=72723\;€$$

were spent for the detection of a so far unknown subject with probable glaucoma (high risk glaucoma suspect).

**Table 11:** Cost-calculation of the whole study.

logistics			
	Accommodation	18000	
	transportation costs	5000	
	medical fee (reply)	1000	
	IT-Administration	6000	
	Advertising	4000	
Σ			34000
examination devices			
	Canon CGI	20000	
	Pachymetry	12000	
	HRT/IOP/FDT	60000	
Σ			92000
personal expenses			
	employees (non-productive work)	141000	
	medical staff (company)	83000	
	company medical officers	25000	
examiners (on-site)			
	Postgraduates	8000	
	doctor-in-training	60000	
Logistics	assessment (consultant)	56000	
	Statistics	8000	
	data bank management	4000	
Σ			385000
Σ Total			511000

## Discussion

A number of studies have been performed in several countries in order to determine the prevalence of glaucoma in the population [6,7,23–29]. Due to differences in the definition of the disease, prevalence estimates cannot be compared easily. To avoid progression any individual with glaucoma needs to be identified and referred to a specialist for further diagnostics and treatment.

In this study, a glaucoma screening was performed by occupational health medicine personnel in a setting of the chemical industries as add-on to routine eye examinations. In an off-site evaluation a glaucoma expert identified 13 probable and 98 possible glaucoma suspects among 4167 active workers between 40 and 65 years of age. Thus, prevalence of all glaucoma suspects was 2.66%. Among the 13 subjects that were classified as probable glaucoma suspects, six were already treated for glaucoma but 7 had not been detected so far. This corresponds well with previous findings that 50% of glaucomas are untreated [6]. We calculated that 595 individuals have to be screened to detect one probable glaucoma case who has not been diagnosed as having glaucoma. Based on the algorithm, the number needed to treat was calculated as 695. To detect one additional glaucoma suspect, we estimated a number needed to treat of 41 based on the expert’s decision and 39 if the algorithm is applied.

We developed a screening algorithm describing the glaucoma diagnosis process, measured the diagnostic accuracy of the algorithm in comparison to the glaucoma expert and compared additionally three straight forward screening approaches based on IOP alone and combinations of IOP and FDT results.

All models showed a diagnostic capacity beyond chance demonstrated by statistically significantly elevated odds ratios. However, the kappa statistics were very low for the simple procedures (cp S1 Appendix for how to evaluate kappas).

To better understand the reliability of the different approaches we calculated additional statistics such as the likelihood ratios describing diagnostic accuracy.

Positive and negative likelihood ratios describe the discriminatory properties of positive and negative test results, respectively. Positive likelihood ratios state how many times more likely particular positive test results are in patients with disease than in those without disease. Negative likelihood ratios state how many times less likely particular negative test results are in patients with disease than in those without disease.

Positive likelihood ratios and inverse negative likelihood ratios above 10 (the latter is equivalent to negative likelihood ratios below 0.1) have been noted as providing convincing diagnostic evidence, whereas those above 5 (below 0.2) give strong diagnostic evidence [30].

High values in positive likelihood ratios and low values in negative likelihood ratios show the correlation of a test model in comparison with the prevalences before the test and are the basis for the calculation of post-test-prevalences in the following calculations.

The pre-test prevalence was given by the ophthalmologist’s decision, it was p = 0.0266. In case of a positive test of the algorithm (glaucoma suspect), the probability p was 0.0279 (Table 6), the positive likelihood ratio 147.8 and the negative likelihood ratio 0.16. We calculated the pre-test odds, post-test odds and the post-test-prevalence as follows (see S1 Appendix):

$$odds_{pre}=\frac{p}{1-p}=\frac{0.0266}{1-0.0266}=0.0273$$

$$odds_{post}=LR_{pos}\cdot odds_{pre}=147.8\cdot 0.0273=4.039$$

$$p_{post}=\frac{odds_{post}}{1+odds_{post}}=\frac{4.039}{1+4.039}=0.802$$

In case of a negative screening result (negative likelihood ratio = 0.16, Table 6), the calculation is as follows:

$$odds_{post}=LR_{neg}\cdot odds_{pre}=0.160\cdot 0.0266=0.004256$$

$$p_{post}=\frac{odds_{post}}{1+odds_{post}}=\frac{0.004256}{1+0.004256}=0.00424$$

This means that in case of a positive screening result the prevalence that there is glaucoma suspect is 0.802 and in case of a negative result the prevalence that there is glaucoma suspect is 0.00424. The screening data generated by the algorithm increases substantially our information about glaucoma suspects. This pronounced discriminatory power reflects the good agreement of the screening algorithm with the ophthalmologist’s decision.

On the basis of these results–and not only because of the high sensitivity and specificity as well as the positive and negative predictive values–a high diagnostic accuracy of the screening algorithm is verified. In case of an insufficient simulation, the post-test-prevalences are not really different from the pre-test-prevalences. This may be illustrated by screening model 3, where only the IOD is taken into consideration. A positive test result is given when the IOD lies over 21 mm HG and a negative test result is given, when it is below.

The calculations for these values (compare Table 6) are as follows:

$$odds_{post}=LR_{pos}\cdot odds_{pre}=7.57\cdot 0.0273=0.207$$

$$p_{post}=\frac{odds_{post}}{1+odds_{post}}=\frac{0.207}{1+0.207}=0.171$$

$$odds_{post}=LR_{neg}\cdot odds_{pre}=0.48\cdot 0.0273=0.0131$$

$$p_{post}=\frac{odds_{post}}{1+odds_{post}}=\frac{0.0131}{1+0.0131}=0.013$$

This means that in case of a positive test result (IOD > 21 mm Hg) the probability that there is glaucoma suspect is only 0.207 and in case of a negative result (IOD < 21 mm Hg), the probability that there is a glaucoma suspect is 0.013. In comparison to the pre-test prevalence of 0.0266 this screening model does not increase post-test prevalence in case of a positive result or decrease it in case of a negative value sufficiently. Together with the low accuracy described by the predictive values of only 17% for glaucoma suspects and 2.2% for probable glaucoma these findings demonstrate convincingly that screening method 3 is inappropriate. Screening methods 1 and 2 are only slightly better. Thus, the simple methods 1–3 do not qualify for screening programs.

In a meta-analysis by Tuck and Crick [31] the prevalence data for primary open angle glaucoma (POAG) was taken from eight population surveys. The study found a prevalence of 0.4% in the age-group of 40–54 (taking account of detected glaucoma including all glaucoma suspects with need of treatment). It is tempting to compare this with our results of 91 glaucoma suspects (2.15%) in the same age group. We advise against doing such comparisons because the definition of glaucoma suspects is not standardised across investigations.

In different population based studies, heterogeneous definitions of glaucoma were used depending on the available data and distributions of parameters (Table 12).

**Table 12:** Varying criteria in a diagnosis of glaucoma (according to [32]), complemented by the Tajimi study [33] and [34] and the definition of Foster et al. [35].

Baltimore Eye Survey [6]
Definite, probable, and uncertain classification. Sometimes not quantified, different disc criteria (CDR ≥ 0.8, or difference between OU ≥ 0.3 or 0.4). VF defect not explainable by other causes. No IOP criterion.
**Barbados Eye Study** [36]
VF, optic disc, and ophthalmic examination criteria. Seven combinations possible. Definite: At least on succession 2 abnormal VF together with 2 of 3 of following criteria: CDR ≥ 0.7, asymmetry ≥ 0.2, rim width ≤ 0.1, notching, disc hemorrhage. If not: suspect. IOP no criterion.
**Beaver Dam Eye Study** [37]
At least two of the following criteria: VF defect not explainable by other causes, CDR ≥ 0.8 or an asymmetry in CDR ≥ 0.2, IOP ≥ 22 mm Hg, or IOP-lowering treatment.
**Blue Mountains Eye Study** [24]
Glaucomatous VF defect not explainable by other causes, combined with VCDR ≥ 0.7, or asymmetry in VCDR between both eyes ≥ 0.3.
**Egna-Neumarkt Study** [26]
At least 2 of the following criteria with open angle: Glaucomatous VF defect, IOP ≥ 22 mm Hg and 1 of the following disc criteria: CDR ≥ 0.7, or asymmetry > 0.2, or difference in VCDR and HCDR > 0.2, or notching, or disc hemorrhage, or excavation reaching disc margin.
**Framingham Study** [38]
VF defect not explainable by other cases (only in selected part of the population), combined with VCDR ≥ 0.6, or asymmetry in VCDR between both eyes ≥ 0.2.
**Melbourne Visual Impairment Project** [39]
No strict criteria due to uncertainty of diagnostic criteria. Panel discussion with 6 ophthalmologists grading in none, possible, probable, or definite POAG. Criteria: past POAG history, IOP > 21 mm Hg, VF defect including enlarged blind spot, CDR ≥ 0.7, or asymmetry ≥ 0.3.
**Ponza Glaucoma Study** [25]
Glaucomatous VF defects and 1 of the following criteria: IOP > 20 mm Hg, CDR ≥ 0.5, or asymmetry ≥ 0.2. Suspect if questionable VF loss.
**Rotterdam Study** (2000 criteria) [32]
If present in at least 1 eye with open angle and no history or sign of secondary glaucoma. No IOP criteria. Definite OAG: GVFD combined with at least possible GON: VCDR ≥ 0.7, or asymmetry between both eyes ≥ 0.2, or a minimal rim width < 0.1.
Probable OAG: (1) GVFD without possible GON or (2) absence of GVFD or of any VF test with probable GON: VCDR ≥ 0.9, or asymmetry ≥ 0.3, or minimal rim width < 0.05. Possible OAG: possible GON and no GVFD.
**Tajimi Study** [33] and [34]
Glaucoma suspect: CDR ≥ 0.7 and < 0.9, a rim width in the superior or inferior portion of ≥ 0.05 and < 0.1, a difference in the VCDR ≥ 0.2 and < 0.3 between both eyes, and a nerve fibre layer defect, but the HFA results were unreliable or unavailable or did not show a compatible defect.
**Definition and classification of glaucoma in prevalence studies** [35]
Category 1 diagnosis (structural and functional evidence) Eyes with a CDR or CDR asymmetry >97.5th percentile for the normal population, or a neuroretinal rim width reduced to < 0.1 CDR (between 11 to 1 o’clock or 5 to 7 o’clock) that also showed a definite visual field defect consistent with glaucoma.
Category 2 diagnosis (advanced structural damage with unproved field loss) If the subject could not satisfactorily complete visual field testing but had a CDR or CDR asymmetry > 99.5th percentile for the normal population, glaucoma was diagnosed solely on the structural evidence.
In diagnosing category 1 or 2 glaucoma, there should be no alternative explanation for CDR findings (dysplastic disc or marked anisometropia) or the VF defect (retinal vascular disease, macular degeneration, or cerebrovascular disease).
Category 3 diagnosis (Optic disc not seen. Field test impossible). If it is impossible to examine the optic disc, glaucoma is diagnosed if:
(A) The visual acuity < 3/60 and the IOP > 99.5th percentile, or
(B) The visual acuity < 3/60 and the eye shows evidence of glaucoma filtering surgery, or medical records were available confirming glaucomatous visual morbidity.

CDR: cup-disc ratio; GON: glaucomatous optic neuropathy; GVFD: glaucomatous visual field defect; HCDR: horizontal cup–disc ratio; HFA: Humphrey Field Analyzer; IOP, intraocular pressure; OD, right eye; OS: left eye; OU: oculus uterque; VCDR: vertical cup–disc ratio; VF: visual field.

One of the reasons for these varying definitions listed in Table 12 are the different aims of the studies. Foster et al. [35] suggested a model which is based on statistical parameters describing the ONH and the visual field or the medical records if available. Their definition is intended for use in epidemiological research. They pointed out that describing the vertical cup to disc ratio (VCDR) without considering the size of optic nerve head is a potential weakness of the definition.

Independently of the definition of POAG and glaucoma as such in the scientific literature, the criteria for the definition for glaucoma are different, especially for vertical or horizontal cup to disc ratio (CDR). This is the case because a small ONH with a CDR of 0.4 may have glaucoma and a large ONH with a CDR of 0.7 may have no glaucoma [40,41].

When the aim of the study is to identify glaucoma suspects the definition is supposed to be stronger. Since an individual may have glaucoma in a very early stage, especially before manifestation in the visual field, we should take into account some asymmetries in the CDR either horizontal or vertical and in the IOP in the same eye. The aim of our screening is beyond an epidemiological description of our population but intended to be practical. The study was designed for the application without the presence of an ophthalmologist on-site, only with medical assistance staff. Therefore, we could not use a slitlamp for identifying individuals with a risk of primary angle closure glaucoma. This type of glaucoma is very rare in the age below 65 in a European population in contrast to Asian populations [35,42,43].

Comparisons across studies are complicated further by the distinction of certain subtypes of glaucoma. Some of the studies try to deliver a specific definition for primary open angle glaucoma (POAG) and to differentiate from secondary forms such as pigmentary or pseudoexfoliative glaucoma [32]. Such a differentiation is far beyond scope of this study. The aim of this investigation was to find all forms of glaucoma because for the blinding individual the specific subtype is of no relevance at this stage. If a glaucoma suspect was identified in our study, the subject was referred to an ophthalmological specialist who may have initiated further diagnostics for adequate treatment of the actual glaucoma form. Note that the final evaluation for statistical analysis was established by a cross-tabulation (Table 2) in which we contrasted the right and the left eye and evaluated both sides to reach a final classification of the subject.

Other factors that confound epidemiologic findings on glaucoma prevalences are varying age structures across studies. As glaucoma risk increases with age, glaucoma prevalence increases in older populations. In our study only subjects up to 65 years participated.

Since glaucoma is a disease that develops over many years, a diagnosis at a specific moment in an early stage is not possible. Determination of parameters can only describe a certain statistical possibility for the presence of the disease. Therefore, we used the terms “glaucoma suspects” throughout and calculated measures of diagnostic accuracy to evaluate screening procedures.

A systematic review and economic evaluation of screening methods for open angle glaucoma [44] concluded that a “highly specific test is required to reduce large numbers of false-positive referrals”. This supports our view that the simple screening models 1, 2 and 3 are inappropriate. The authors stated that “glaucoma detection can be improved by increasing attendance for eye examination, and improving the performance of current testing by either refining practice or adding in a technology-based first assessment, the latter being the more cost-effective option.” The latter is the approach we have taken in this study. Burr et al. [44] emphasized that “no randomised controlled trials (RCTs) of screening were identified”. We note that this limits all conclusions about the effectiveness of glaucoma screening procedures.

Because no RCTs were found a meta-analysis was undertaken on studies assessing candidate screening tests for detecting open angle glaucoma in persons older than 40 years that reported true and false positives and negatives [45]. The authors described the material identified as “heterogeneous data of limited quality and as such are associated with considerable uncertainty” and stated that “further research is needed to evaluate the comparative accuracy of the most promising tests”.

A Cochrane review [46] of screening methods for prevention of optic nerve damage due to chronic open angle glaucoma reported also that no randomized controlled trials were identified according to their inclusion criteria. The authors concluded that “on the basis of current evidence, population-based screening for chronic OAG cannot be recommended although much can be done to improve awareness and encourage at risk individuals to seek testing.” We believe that our project studying active workers and asking subjects with indications of glaucoma to consult an ophthalmological expert is in line with these recommendations.

The U.S. Agency for Healthcare Research and Quality investigated the comparative effectiveness of screening for glaucoma within the Effective Health Care Program [47]. The authors did not identify any systematic review or study that provided evidence for direct or indirect links between glaucoma screening and visual field loss, visual impairment, optic nerve damage, intraocular pressure, or patient-reported outcomes.

Because of the general lack of evidence for effectiveness we were unable to design our study using well-established screening methods for glaucoma. We suggest our decision tree algorithm as a candidate for further testing as it has shown appropriate diagnostic accuracy in this investigation, in particular in comparison to the simple procedures 1, 2 and 3 based on intraocular pressure and FDT findings only. A major weakness of our study is the missing independent validation of the expert’s classification into “no glaucoma”, “possible glaucoma” and “probable glaucoma” by a second or third glaucoma expert. This would allow a more reliable examination of the usefulness of the algorithm.

Participation rate, realized sex and age distributions, strengths and weaknesses of design and conduct of our study as well as other features of our investigation were discussed elsewhere [8].

About 73,000 € were spent for the detection of a so far unknown subject with probable glaucoma in this study (high risk glaucoma suspect). We note that this refers to an active worker population that must show a sufficient health condition to stay in the job (“healthy worker survivor effect” [48]). In addition, age of the screened workers was between 40 years and 65 years (Table 3) and is rather low in comparison to studies based on the general population. Thus, we hesitate to compare disease prevalences, cost estimates or numbers needed to screen with those derived in other studies that do not describe active worker populations. We like to emphasize that despite these specific circumstances our investigation detected seven high risk glaucoma suspects that were unknown so far. The algorithm was able to identify six of these probable glaucomas.

In summary, our approach and set-up seem to define a feasible procedure to screen for glaucoma suspects in a working population. The examinations have been performed in the company (occupational health department). The evaluation was done later at Johannes Gutenberg University in Mainz by an ophthalmological expert, who has been handed over the data electronically. The developed screening model (algorithm) seems to be applicable in such a population with relatively low age and low glaucoma prevalence and shows an appropriate diagnostic accuracy. The algorithm can be applied when findings from fundus photographies are available. Intraocular pressure failed to qualify as a screening procedure because the sensitivity for probable glaucoma suspects was just about 60% and the positive predicted value was as low as 2.2%. Adding FDT data did not lead to substantially better findings.

The authors are well aware that the developed screening model (algorithm) fits very well in this specific population with this specific ophthalmologist who assessed the fundus photographies. Although the model was constructed for a working population it is supposed to fit in an older population with a higher glaucoma prevalence too. Different experts should validate the screening model not only in the same population but also in different working or general populations with a different composition regarding age and sex.

## Supporting Information

S1 Appendix (DOCX)
