# Supplementary material for: The Evonik-Mainz Eye Care-Study (EMECS): Development of an Expert System for Glaucoma Risk Detection in a Working Population
Source: PLoS One. 2016 Aug 1;11(8):e0158824. doi: 10.1371/journal.pone.0158824 (PMC4968826; doi:10.1371/journal.pone.0158824)
Supplement: S1 Appendix — (DOCX) [file pone.0158824.s001.docx]

Appendix

Definition and relationship among terms used to describe diagnostic accuracy

The following 2x2 table reports on the test results and the true status of N subjects [[11-14](#_ENREF_11)]:

| 2x2 Table on N= a+b+c+d subjects |  | Diagnosis by Expert ("[gold standard](http://en.wikipedia.org/wiki/Gold_standard_(test))") | | Ʃ |
| --- | --- | --- | --- | --- |
|  |  | *positive* | *negative* |  |
| Diagnosis by Algorithm or Screening Procedure  (“test outcome”) | *positive* | a (true positive) | b (false positive, [type I error](http://en.wikipedia.org/wiki/Type_I_and_type_II_errors#False_positive_rate)) | a+b |
|  | *negative* | c (false negative, t[ype II error](http://en.wikipedia.org/wiki/Type_I_and_type_II_errors#False_negative_rate)) | d (true negative) | c+d |
| Ʃ |  | [a+c](http://en.wikipedia.org/wiki/Sensitivity_and_specificity) | [b+d](http://en.wikipedia.org/wiki/Sensitivity_and_specificity) | N |

We used the following terms to measure diagnostic accuracy based on the 2x2 table:

Sensitivity (positive tests among true positives): a/(a+c)

Specificity (negative tests among true negatives): d/(b+d)

Positive Predictive Value (true positives among positive tests): a/(a+b)

Negative Predictive Value (true negatives among negative tests): d/(c+d)

Diagnostic Odds Ratio (cross product ratio, measure of dependence) ad/bc
= (a/c)/(b/d), i.e., the odds of positive tests among true positives divided by the odds of positive tests among true negatives
= (a/b)/(c/d), i.e., the odds of true positives among positive tests divided by the odds of true positives among negative tests

Positive Likelihood Ratio (positive tests among true positives / positive tests among true negatives) = sensitivity / (1 − specificity) = (a/b) / [(a+c)/(b+d)], i.e.,
the odds of true positives among positive tests divided by the marginal odds of true positives (ignoring the test values). Thus, given a positive test result:
post-test odds for true positives = positive likelihood ratio x pre-test odds for true positives

Negative Likelihood Ratio (negative tests among true positives / negative tests among true negatives) = (1 − sensitivity) / specificity = (c/d) / [(a+c)/(b+d)], i.e.,
the odds of true positives among negative tests divided by the marginal odds of true positives (ignoring the test values). Thus, given a negative test result:
post-test odds for true positives = negative likelihood ratio x pre-test odds for true positives

Note that the prevalence of true positives and the odds of true positives are related: prevalence = odds / (1+odds) and odds = prevalence / (1- prevalence)

Positive likelihood ratios and inverse negative likelihood ratios above 10 (the latter is equivalent to negative likelihood ratios below 0.1) have been noted as providing convincing diagnostic evidence, whereas those above 5 (below 0.2) give strong diagnostic evidence [[30](#_ENREF_30)].

The kappa statistic allows us to measure agreement above and beyond that
expected by chance alone [[49](#_ENREF_49),[50](#_ENREF_50)].

| Interpretation of kappa statistics: how strong is the agreement? | |
| --- | --- |
| < 0.00 | Poor |
| 0.00 - 0.20 | Slight |
| 0.21 - 0.40 | Fair |
| 0.41 - 0.60 | Moderate |
| 0.61 - 0.80 | Substantial |
| 0.81 - 1.00 | Almost Perfect |

**References**

11. Altman DG, Bland JM (1994) Diagnostic tests. 1: Sensitivity and specificity. BMJ 308: 1552.

12. Altman DG, Bland JM (1994) Diagnostic tests 2: Predictive values. BMJ 309: 102.

13. Deeks JJ (2001) Systematic reviews in health care: Systematic reviews of evaluations of diagnostic and screening tests. BMJ 323: 157-162.

14. Zwinderman AH, Bossuyt PM (2008) We should not pool diagnostic likelihood ratios in systematic reviews. Stat Med 27: 687-697.

30. Altman DG, Bland JM (1994) Statistics notes: diagnostic tests 2: predictive values. BMJ 309: 102.

49. Landis JR, Koch GG (1977) The measurement of observer agreement for categorical data. Biometrics 33: 159-174.

50. McGinn T, Wyer PC, Newman TB, Keitz S, Leipzig R, et al. (2004) Tips for learners of evidence-based medicine: 3. Measures of observer variability (kappa statistic). Canadian Medical Association Journal 171: 1369-1373.
